# FlowRoI: fast optical-flow-based Roi extraction for high-throughput immune cell image compression

**DOI:** 10.1038/s44303-026-00178-3

**Published:** 2026-07-11

**Authors:** Xiaowei Xu, Justin Sonneck, Hongxiao Wang, Roman Burkard, Hendrik Wöhrle, Anton Grabmaier, Matthias Gunzer, Jianxu Chen

**Affiliations:** 1 Leibniz-Institut für Analytische Wissenschaften- ISAS- e.V., Dortmund, Germany; 2 Guangdong Provincial People’s Hospital (Guangdong Academy of Medical Sciences), Southern Medical University, Guangzhou, China; 3 Faculty of Computer Science, Ruhr-University Bochum, Bochum, Germany; 4 Academy for Multidisciplinary Studies, Capital Normal University, Beijing, China; 5 Department of Electrical Engineering and Information Technology, University of Duisburg-Essen, Duisburg, Germany; 6 Fraunhofer Institute for Microelectronic Circuits and Systems, Duisburg, Germany; 7 Institute for Experimental Immunology and Imaging, University Hospital, University of Duisburg-Essen, Essen, Germany

**Keywords:** Biological techniques, Computational biology and bioinformatics, Engineering, Mathematics and computing

## Abstract

Autonomous migration is central to neutrophil function and diverse disease processes. ComplexEye, our recently introduced multi-lens array microscope, enables high-throughput live-cell video acquisition for routine quantification of autonomous motility. However, such platforms generate data at extreme scale, creating substantial challenges for storage and transmission. Here we present FlowRoI, a fast, training-free framework for region-of-interest (RoI) extraction and RoI-aware compression in immune cell migration studies. FlowRoI computes optical flow between consecutive frames and derives RoI masks that effectively capture migrating cells in the evaluated dataset. Each frame and its RoI mask are then jointly encoded using JPEG2000 to achieve efficient, cell-focused compression. FlowRoI is computationally lightweight, operating at approximately 30 frames per second on a laptop with an Intel i7-1255U CPU—comparable to standard JPEG2000. At matched peak signal-to-noise ratio, FlowRoI achieves approximately 2 × higher compression rates than standard JPEG2000 in the evaluated dataset while preserving higher image quality in cellular regions. To assess downstream impact, we evaluated cell instance segmentation as a representative task. At comparable segmentation accuracy in our experiments, FlowRoI provides approximately 2 × higher compression efficiency. FlowRoI requires only a few hyperparameters and shows stable performance across the tested settings, facilitating practical parameter selection. Together, these findings demonstrate the feasibility of FlowRoI as a computationally efficient, domain-oriented approach for task-aware compression in bright-field neutrophil migration imaging. Its applicability to other cell types, imaging modalities, and experimental settings remains to be systematically evaluated.

## Introduction

Autonomous migration is essential for the function of immune cells and plays a central role in numerous diseases^1–4^. Among these cells, neutrophils—the most abundant leukocytes in human blood—act as critical first responders^5^. They rapidly migrate into inflamed tissues within minutes, enabling immediate host defense^6^. However, this same migratory capacity can be detrimental: neutrophil infiltration not only protects against infection but may also aggravate pathology. For example, their recruitment into tumors correlates with poor prognosis^7^, and excessive migration into ischemic heart^8^ or brain^9^ tissue exacerbates sterile injury. Thus, autonomous migration represents a double-edged sword, capable of conferring both protection and harm.

Decades of work have uncovered molecular mechanisms that govern neutrophil migration^10^. The bacterial peptide fMLP, for instance, potently induces chemotaxis toward infection^11^, yet structurally similar ligands originating from host mitochondria can redirect neutrophils into sterile inflammatory sites. Depending on context, such migration may support tissue repair—e.g., by promoting revascularization—or worsen pathology, as observed in stroke and cancer. Consequently, distinguishing and selectively modulating these divergent migratory programs is of considerable therapeutic interest. However, given the diversity of chemotactic ligands, predicting modulatory effects computationally is nearly impossible, necessitating systematic high-throughput experimental approaches. High-throughput migration assays enable parallel, large-scale functional profiling, facilitating the discovery of selective modulators that might, for example, prevent neutrophil entry into tumors while preserving antimicrobial defense.

To address this need, we recently introduced ComplexEye^12^, a multi-lens video microscope that integrates 16 aberration-corrected glass lenses, each equipped with an individual detector and illumination path. This configuration allows simultaneous imaging of 16 wells of a 96-well plate, or 64 wells of a 384-well plate, at one frame every 8 seconds. ComplexEye enables energy-efficient, high-throughput migration analysis across hundreds of conditions. We demonstrated that the system can process multiple clinical samples in parallel and can screen 1000 compounds to identify 17 modulators of human neutrophil migration within four days—a task that would require approximately 60 times longer using a conventional video microscope.

However, high-throughput immune cell migration imaging generates data at an unprecedented scale, intensifying the long-standing storage challenge in microscopy. Over the past decade, the widespread adoption of digital imaging and advances in multidimensional microscopy have driven explosive data growth, with many core facilities now producing petabytes of data annually^13–15^. A single high-content screening experiment may generate hundreds of thousands of images^16^. In the ComplexEye platform, a one-hour experiment produces 28,800 images (about 16 GB). In industrial-scale settings, yearly data volumes easily reach the petabyte range^14^. The storage and transmission of such massive datasets are both time-consuming and costly, underscoring the urgent need for efficient image compression tailored to high-throughput migration analysis.

Here, we introduce FlowRoI, a fast optical-flow-based region of interest (RoI) extraction framework designed for high-throughput image compression in immune cell migration studies. FlowRoI first computes optical flow between adjacent frames and derives RoI masks that capture the majority of all migrating cells. The raw image and the corresponding RoI mask are then jointly encoded using JPEG2000 to enable RoI-based compression. FlowRoI operates efficiently, achieving about 30 frames per second on a standard laptop with an Intel i7-1255U CPU. In cell regions, FlowRoI yields higher peak signal-to-noise ratio (PSNR), and in the evaluated dataset, it achieves approximately 2 × higher compression rates than standard JPEG2000 at matched PSNR. Unlike conventional RoI-based compression methods that rely on predefined or annotated regions, FlowRoI derives task-relevant regions directly from motion information, enabling a fully training-free and data-adaptive pipeline.

To validate downstream information preservation, we used cell instance segmentation as a representative and foundational analysis task, because accurate cell localization and boundary delineation are prerequisites for many higher-level migration analyses, including tracking, motility estimation, and morphology quantification. At comparable segmentation performance, it achieves approximately 2 × higher compression rates in our experiments. Importantly, FlowRoI is training-free, requires only a small set of hyperparameters, and remains stable across a wide range of parameter choices. Although a small number of cells are not captured in the RoI masks, we observe no substantial degradation in downstream segmentation performance under the evaluated setting. Finally, we provide an analysis of key hyperparameters, offering guidance for potential deployment in high-throughput imaging workflows.

Because the evaluation is conducted on a limited dataset without an independent test set, the conclusions of this study are restricted to the examined imaging conditions, namely bright-field microscopy of neutrophil migration, and should not be interpreted as evidence of generalization across different cell types, imaging modalities, or experimental settings.

Accordingly, FlowRoI should be interpreted as a domain-specific, motion-driven preprocessing framework for sparse cell-migration imaging rather than a generally validated compression framework for microscopy data.

Existing approaches to image and video compression can be broadly categorized into general-purpose codecs, RoI based compression methods, and learning-based compression models. Classical RoI-based approaches typically rely on predefined regions or annotations to guide bit allocation, while learning-based methods often require large amounts of training data and computational resources. In contrast, our approach focuses on a task-driven setting in which dynamically relevant regions are identified directly from motion cues, without requiring supervision. Rather than introducing a new compression codec, FlowRoI provides a lightweight and training-free mechanism to adapt existing compression schemes to task-relevant regions in cell migration imaging.

The main contributions of this work are as follows:

- We formulate task-aware compression for cell migration imaging as a problem of prioritizing dynamically relevant regions, highlighting the role of motion as a proxy for biological importance.
- We propose FlowRoI, a training-free RoI extraction framework based on optical flow that captures dynamic cellular regions without requiring annotated data or learned models.
- We integrate the proposed RoI extraction with JPEG2000 RoI coding to enable efficient compression while preserving information relevant to downstream analysis.
- We provide a task-aware evaluation by combining reconstruction quality and downstream segmentation performance, demonstrating consistent improvements under the evaluated setting.

## Results

### Experimental setting

We evaluated FlowRoI using six ComplexEye videos comprising a total of 2628 2D images.

All videos were acquired under the same bright-field imaging setting and contain primary human neutrophils; therefore, the experimental results should be interpreted within this specific imaging and biological context.

These videos depict primary human neutrophils isolated from peripheral blood, and the dataset is publicly available online^17^. Additional information regarding sample preparation and ethical approvals is provided in the ComplexEye paper^12^, which includes the corresponding ethics declarations and informed consent.

For downstream analysis, we used Cellpose-SAM as the instance-segmentation backbone (detailed training procedure is provided in the Methods Section). As a compression baseline, we report frame-wise JPEG2000 following common practice in biomedical imaging, where still-image compression is typically used for storage and archiving. Although modern video codecs can also exploit temporal redundancy, their effectiveness in microscopy imaging may depend on imaging conditions, motion characteristics, and codec design. Therefore, we do not make general claims that video codecs are limited for microscopy compression; instead, we restrict our comparison to JPEG2000-based still-image compression under the present experimental setting. In this work, we focus on a lightweight RoI-oriented preprocessing strategy for sparse immune-cell migration scenes rather than a comprehensive evaluation of contemporary video compression methods. Evaluation metrics include PSNR measured within true cellular regions, and instance-segmentation precision, recall, and F1-score at an IoU of 0.5. Full implementation, annotation procedures, and metric definitions are provided in the Methods Section.

### Overall performance

**Fig. 1:**
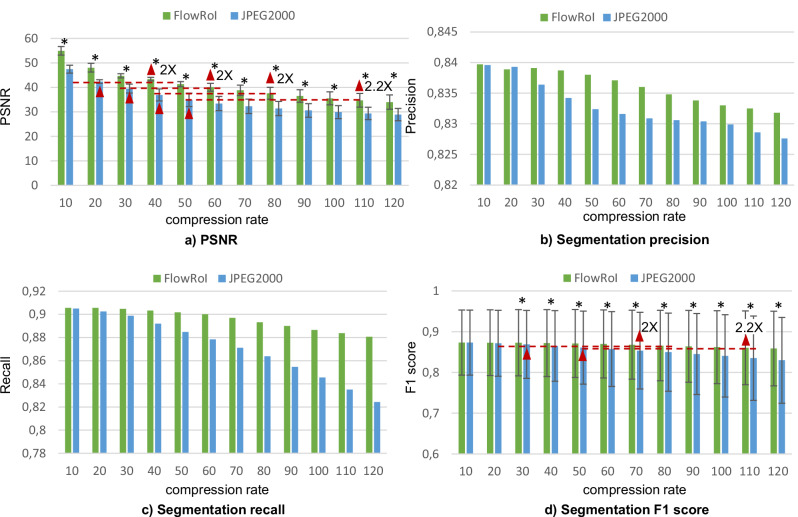
Comparison of FlowRoI and JPEG2000 in image quality and segmentation performance. **a** PSNR comparison across compression rates. **b** Segmentation precision of FlowRoI and JPEG2000. **c** Segmentation recall across varying compression rates. **d** Segmentation F1 scores showing overall segmentation accuracy. Bars indicate mean performance across the six evaluated videos, and error bars are shown for representative metrics (PSNR and F1 score) and represent standard deviation across videos. Statistical significance between FlowRoI and JPEG2000 was assessed using paired Wilcoxon signed-rank tests across videos (*p < 0.05).

Figure 1 compares FlowRoI and JPEG2000 in terms of image quality and downstream instance segmentation across a range of compression rates. PSNR is computed within ground-truth cell regions, reflecting reconstruction fidelity in task-relevant areas. All quantitative results are reported as the mean performance across the six videos, and dispersion measures are provided to illustrate inter-video variability. Both methods show gradual declines in PSNR and segmentation performance as compression increases, with sharper degradation at higher compression levels. Importantly, the performance drop occurs later for FlowRoI than for JPEG2000, suggesting that FlowRoI preserves task-relevant information more effectively under stronger compression in this experimental setting. The fine-tuned Cellpose-SAM model achieved a precision above 0.80, consistent with the performance reported in the original publication^18^—confirming that our retraining procedure was correctly performed. Across the evaluated compression rates (Fig. 1a), FlowRoI consistently achieves higher PSNR than JPEG2000. At equal PSNR, FlowRoI provides approximately 2.0–2.2 × higher compression efficiency in the evaluated dataset. Similarly, at comparable segmentation precision, FlowRoI provides approximately 2.0–2.2 × higher compression efficiency in our experiments.

To further assess evaluation fairness beyond cell-region metrics, we additionally evaluated full-image reconstruction quality using global PSNR and SSIM measurements (Fig. S1). Compared with the cell-region evaluation, improvements in full-image metrics are smaller, which is expected because most pixels correspond to background regions that are more aggressively compressed in the RoI-based framework. Nevertheless, FlowRoI achieves consistently competitive or higher full-image PSNR and SSIM under most evaluated compression settings, indicating that prioritizing biologically relevant cellular regions does not substantially compromise overall image fidelity. Interestingly, at low compression settings (e.g., 10 × compression), JPEG2000 achieves slightly higher full-image quality, likely because the available bitrate is already sufficient to preserve both cellular and background regions without strong prioritization. Under stronger compression, however, the RoI-aware strategy of FlowRoI becomes increasingly beneficial for preserving task-relevant cellular structures while maintaining competitive global image quality.

**Fig. 2:**
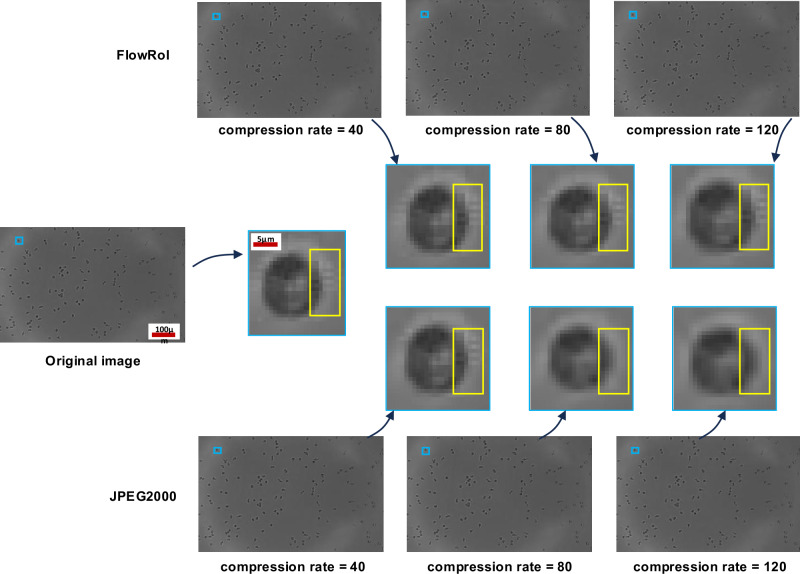
Qualitative comparison of FlowRoI and JPEG2000 at different compression rate.

A qualitative comparison is shown in Fig. 2. At the full-image scale, both methods appear visually similar. However, after zooming into local regions (highlighted in yellow), FlowRoI preserves fine cellular texture and boundary morphology across all compression rates, even at a compression rate of 120. JPEG2000 preserves similar detail only at a compression rate of 40; at higher rates (80 and 120), fine structures are blurred or lost.

### Parameter effects on RoI masks and coverage rates

**Fig. 3:**
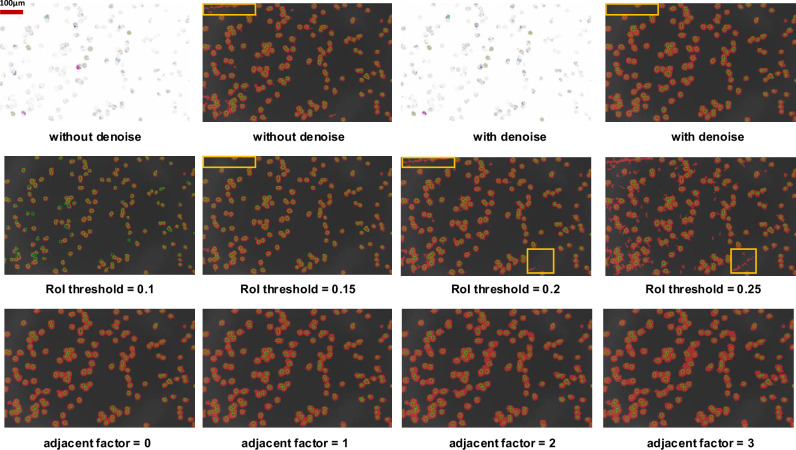
Qualitative intermediate outputs with different settings of denoising, RoI threshold, and adjacent factors. After applying denoising, several scattered and irregular artifacts (highlighted in yellow) are effectively removed, resulting in cleaner RoI masks without affecting the main cell regions.

Figure 3 qualitatively illustrates the intermediate outputs under different settings of the denoising module, RoI threshold, and adjacent factor. Denoising effectively removes scattered and irregular artifacts (highlighted in yellow), yielding cleaner RoI masks while preserving the main cell regions. In practice, occasional false RoIs caused by noise or imaging artifacts were observed, as shown in orange rectangles. However, these regions were not segmented as cells in the downstream analysis. This illustrates that FlowRoI may overestimate regions of interest at the compression stage without adversely affecting cell-level analysis. Increasing the RoI threshold results in larger RoI masks and increased noise due to a greater number of pixels being classified as motion. Likewise, a larger adjacent factor produces larger masks, as motion estimated over more distant frames is typically stronger than that between adjacent frames.

**Fig. 4:**
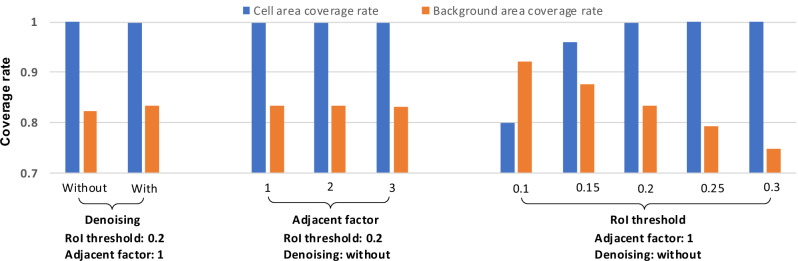
Cell and background area coverage rates under different parameter settings. Cell area coverage rate = 1 means the extracted ROI regions cover all the pixels belonging to cells, therefore indicating the effectiveness of the ROI detection step. When the cell area coverage rate is close to 1 and the the background coverage rate is much lower than one, it represents the situation of a conservative ROI masking.

Cell and background area coverage rates under different parameter settings are shown in Fig. 4, where the effects of denoising, the adjacent factor, and the RoI threshold are analyzed. Note that the cell area coverage rate is defined as the mean ratio between the area of the cell mask covered by all RoI regions and the total area of the corresponding cell mask. The background area coverage rate is defined as the mean ratio between the area of the background covered by non-RoI regions and the total area of the corresponding background region. Compared with the setting without denoising, enabling denoising results in a slightly lower cell area coverage rate and a higher background area coverage rate. A similar trend is observed when varying the adjacent factor, where only minor changes in coverage rates occur. In contrast, the RoI threshold has a more pronounced impact. A small RoI threshold of 0.1 leads to a relatively low cell area coverage rate and a higher background area coverage rate. As the RoI threshold increases, the cell area coverage rate improves while the background area coverage rate decreases. When the RoI threshold exceeds 0.2, the cell area coverage rate saturates and remains nearly constant, whereas the background area coverage rate continues to decrease. This behavior can be explained by the fact that a larger RoI threshold causes more regions to be classified as RoIs. Once the cell regions are fully covered, additional RoI regions increasingly occupy background areas, thereby reducing the background area coverage rate.

### Cell missing cases

**Fig. 5:**
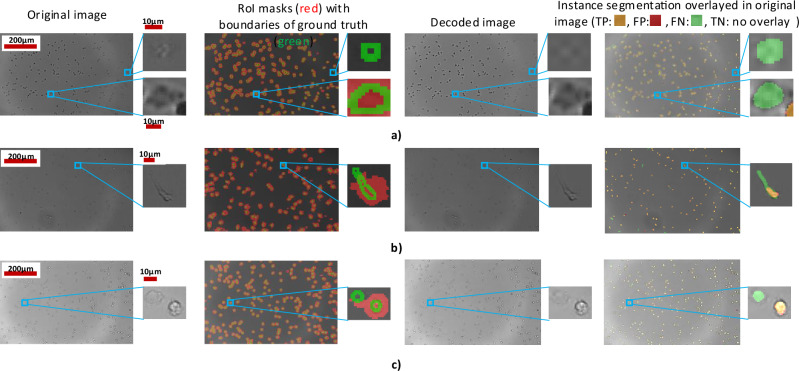
Examples of cell-missing cases under FlowRoI compression. These cases are rare (approximately 0.026% of all instances) and are primarily associated with low-contrast or weakly motile cells. **a** A dim and low-contrast cell missed by RoI but detectable in the original image. **b** A long, elongated neutrophil partially excluded from the RoI. **c** A morphologically atypical cell with weak boundaries not fully captured. TP true positive, FP false positive, FN false negative, and TN true negative.

Although FlowRoI aims to cover all cells while keeping RoI size minimal, some cells may still be missed (Fig. 5). These missed cells typically exhibit very low contrast and appear similar to the background, resulting in weak motion flow and exclusion from RoI masks. During compression, such regions are encoded with the same priority as the background, further reducing their visibility after decoding. Consequently, these cells may not be recognized during instance segmentation. Notably, even in the original uncompressed images, such low-contrast cells are not reliably detected by Cellpose-SAM. For example, as shown in the first row of Fig. 5, the leftmost missed cell has higher contrast than the others but still fails to be segmented correctly. To further quantify these failure cases, we observe that missed detections occur in a very small fraction of instances (107 out of 409,968 total cell instances, approximately 0.026%). This indicates that such cases are rare under the evaluated setting. From a quantitative perspective, these missed cells contribute minimally to the aggregate segmentation metrics, as they represent a negligible proportion of the overall dataset. Consistently, we do not observe substantial degradation in the reported precision, recall, or F1-score. From a biological perspective, since the vast majority of migrating cells are successfully captured, the overall statistical characterization of cell behavior is expected to remain largely unchanged in this setting. However, we note that the potential impact on higher-level motility metrics (e.g., trajectory-based analysis) is not explicitly evaluated in this study and remains an important direction for future work. Importantly, the numerator and denominator are defined consistently at the per-frame instance level, with each cell instance in each frame counted separately.

### Hyperparameter discussion

**Fig. 6:**
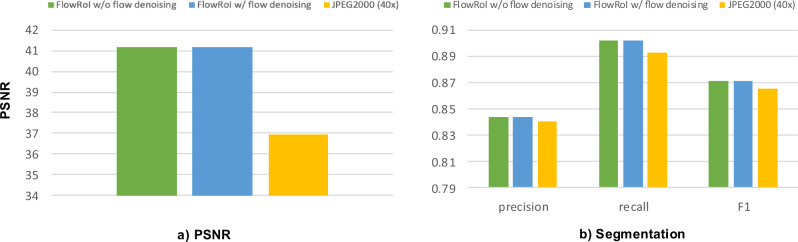
Effect of denoising on image quality and segmentation performance. **a** PSNR comparison with and without denoising, compared to JPEG2000 at 40 × compression. **b** Segmentation precision, recall, and F1 score under the same settings, showing minimal differences between denoised and non-denoised FlowRoI. Other hyperparameters: RoI threshold 0.2, adjacent factor 0, scaling factor 5, compression rate 40. Bars indicate mean performance across the six evaluated videos.

Figure 6 shows the effect of denoising. FlowRoI with and without denoising performs similarly, as the removed noise components are minor. Both versions show better performance than JPEG2000 under this evaluation setting. We also observe that denoising leads to only marginal improvements in image quality and segmentation performance. This is primarily because noise-affected regions occupy only a small fraction of the images, limiting their overall impact. As illustrated in Fig. 3, these noise regions can be effectively suppressed (highlighted by the orange rectangles). Although the quantitative improvement is small, denoising is generally recommended, as it improves the stability of the RoI extraction process.

**Fig. 7:**
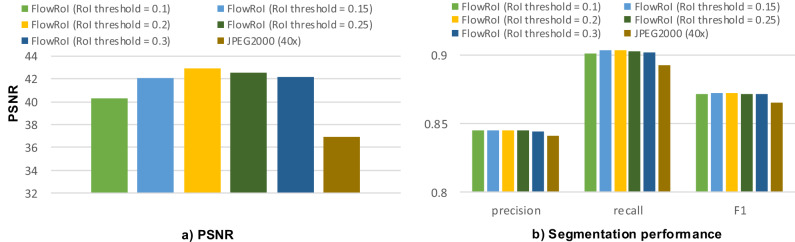
Effect of the RoI threshold on image quality and segmentation performance. **a** PSNR comparison across different RoI thresholds and JPEG2000 at 40 × compression. **b** Segmentation precision, recall, and F1 score for the same settings, showing relatively stable performance once thresholds reach 0.2. Other parameters: denoising 1, adjacent factor 0, scaling factor 5, compression rate 40. Bars indicate mean performance across the six evaluated videos.

Figure 7 illustrates performance at different RoI thresholds. PSNR exhibits an inverse-U trend, peaking at a threshold of 0.2. Instance-segmentation performance shows a different pattern: it increases from thresholds of 0.1 to 0.2, but remains nearly constant for larger values. A likely explanation is that at low thresholds (e.g., 0.1), some cells are not fully included in the RoI (Fig. 3), reducing both PSNR and segmentation accuracy. As the threshold increases, more cells are covered—even if only partially—improving both metrics. When thresholds become large, RoI masks grow too large, leaving fewer bits to encode each RoI pixel and decreasing PSNR. However, no substantial degradation in segmentation performance is observed, likely because the slight blurring affects only a small fraction of the image and has limited influence on IoU-0.5 segmentation in this setting.

**Fig. 8:**
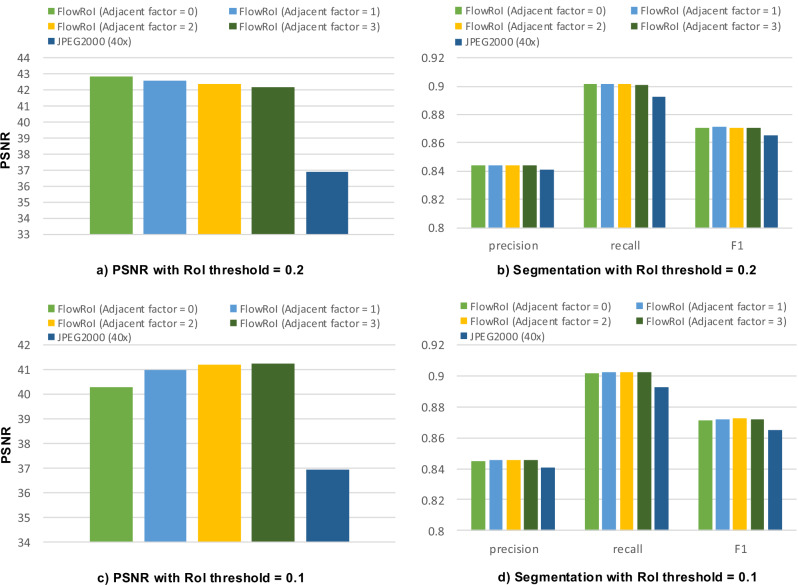
Effect of the adjacent factor on image quality and segmentation performance. **a** PSNR at RoI threshold 0.2 for different adjacent factors. **b** Segmentation precision, recall, and F1 at RoI threshold 0.2. **c** PSNR at RoI threshold 0.1 across adjacent factors. **d** Segmentation performance at RoI threshold 0.1. Other parameters: denoising 1, scaling factor 5, compression rate 40. Bars indicate mean performance across the six evaluated videos.

Figure 8 shows the influence of the adjacent factor. With a high RoI threshold (0.2), increasing the adjacent factor reduces PSNR and segmentation performance by adding excess background to RoI masks. With a low RoI threshold (0.1), increasing the adjacent factor improves both PSNR and segmentation performance by compensating for missed cells via temporal integration.

**Fig. 9:**
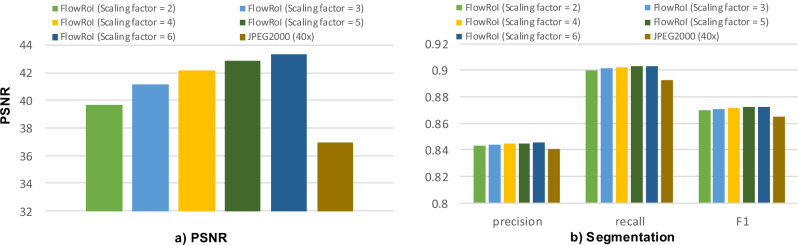
Effect of the scaling factor on image quality and segmentation performance. **a** PSNR across different scaling factors compared with JPEG2000 at 40 × compression. **b** Segmentation precision, recall, and F1 score under the same settings, showing improved image quality but stable segmentation once scaling is sufficiently large. Other parameters: denoise 1, RoI threshold 0.2, adjacent factor 0, compression rate 40. Bars indicate mean performance across the six evaluated videos.

The impact of the scaling factor is shown in Fig. 9. Higher scaling factors increase both PSNR and segmentation performance, as expected. However, PSNR increases more than segmentation accuracy, because once image quality surpasses the threshold required for robust segmentation, further enhancement yields diminishing returns.

## Discussion

We presented FlowRoI, a fast optical-flow-based RoI extraction method for high-throughput image compression in immune cell migration analysis. By leveraging motion cues between adjacent frames, FlowRoI identifies RoIs that cover the majority of all migrating cells while keeping RoI size compact. Combined with JPEG2000 RoI compression, FlowRoI achieves around 2 × higher compression at equal PSNR and about 2 × higher compression at equal segmentation performance compared with standard JPEG2000. FlowRoI runs extremely fast (30 FPS on an Intel i7-1255U laptop CPU), is training-free, requires only a few hyperparameters, and exhibits relatively stable performance across parameter settings. Its simplicity and computational efficiency make FlowRoI a promising domain-oriented approach for reducing storage requirements in high-throughput bright-field neutrophil migration imaging while preserving features relevant to downstream analysis in the evaluated setting.

Across the tested settings, FlowRoI shows relatively stable performance in preserving information relevant to the evaluated image-quality and segmentation metrics. Changes in hyperparameters—such as the use of denoising, the choice of RoI threshold, or the adjacent factor—lead to only minimal differences in segmentation performance, indicating that the core cell structures required for downstream biological analysis are well preserved. The small subset of cells that are not fully included in the RoI masks typically consists of biologically challenging cases, such as extremely dim, weakly motile, or morphologically atypical neutrophils. These missed cells are expected to contribute only minimally to population-level migration statistics under the evaluated setting. Consistent with the quantitative analysis in the results section, these missed instances account for only a very small proportion of all cells (approximately 0.026%), and therefore have limited impact on aggregate evaluation metrics. The impact on higher-level motility readouts, such as persistence or trajectory-based metrics, was not evaluated in this study and should be assessed in future work. Moreover, once the primary cell bodies are captured, slight variations in RoI extent or local image detail do not alter cell identification or mask-level boundaries at the IoU=0.5 level. Together, these observations indicate that FlowRoI maintains the integrity of key biological signals across a broad hyperparameter range, supporting its potential use in large-scale migration assays without extensive tuning.

RoI-based image compression has attracted considerable interest over the past decades. Table 1 summarizes comparisons with related methods. FlowRoI requires no training, runs quickly (0.14 s per case on an Intel i7-1255U CPU using a single thread; JPEG2000 requires 0.09 s under the same conditions), and offers substantial performance gains over JPEG2000. With multi-threading, FlowRoI reaches approximately 30 FPS on the same CPU. In contrast, methods such as^19^ require training with support vector machine classifiers, while^20^ uses Swin Transformer blocks for RoI encoding, requiring large training datasets and GPU resources. Many recent approaches rely on deep learning, achieving strong PSNR improvements but at the cost of complex training procedures and limited deployability due to GPU dependencies. In real-world applications, ease of deployment and processing speed are equally critical. FlowRoI satisfies these requirements but is tailored primarily for microscopy images with sparse, moving targets. It may generalize to similar applications, but its applicability is narrower than that of deep-learning-based compression methods designed for diverse image types.

**Table 1:** Comparison of FlowRoI with representative RoI-based and learning-based compression methods

Method	Training	Annotation	Platform	RoI-based	Task-aware	Notes
RoI compression^19^	Yes	Yes	CPU	Yes	No	Predefined regions
Swin-based RoI compression^20^	Yes	Yes	GPU	Yes	No	Deep model, high complexity
Variable-rate RoI compression^25^	Yes	Yes	GPU	Yes	No	Learned bit allocation
Deep image compression^26^	Yes	No	CPU/GPU	No	No	General-purpose compression
Content-weighted compression^27^	Yes	No	GPU	Yes	No	Learned saliency
Saliency-based compression^28^	Yes	Yes	GPU	Yes	Partial	Requires segmentation
**FlowRoI (ours)**	No	No	CPU	Yes	Yes	Motion-driven, real-time

This study has several limitations. First, the evaluation was conducted on a single bright-field neutrophil migration dataset comprising six videos, and the segmentation model was trained and evaluated on different frames from the same sequences. Therefore, the reported results primarily reflect within-sequence generalization under the examined imaging conditions rather than broad generalization across independent datasets, cell types, or microscopy modalities. Second, the current evaluation focuses mainly on reconstruction quality and downstream instance segmentation performance. Higher-level biological analyses, such as cell tracking, trajectory continuity, and motility quantification, were not explicitly evaluated and remain important directions for future work. Third, the present study uses JPEG2000 as a controlled RoI-compression framework to evaluate the proposed motion-driven RoI extraction strategy. Direct comparisons with modern video codecs or learning-based compression methods were beyond the scope of this work. Finally, FlowRoI is primarily designed for sparse migration-imaging scenarios in which motion provides a reliable cue for identifying biologically relevant regions. Its applicability to densely structured scenes, adherent-cell imaging, or microscopy modalities with substantially different contrast characteristics requires further investigation.

FlowRoI is not intended to replace modern image or video codecs. Rather, it provides a lightweight motion-driven RoI extraction mechanism that can be combined with existing compression frameworks. In the current implementation, the extracted RoI maps are integrated with JPEG2000 RoI coding because of its native support for region-prioritized encoding. Extending the proposed strategy to modern video codecs or learned compression frameworks represents an important direction for future work.

## Methods

### Imaging system and data acquisition

All videos were acquired using the ComplexEye platform^12^, a multi-lens, high-throughput microscope designed for parallel live-cell imaging in standard multi-well plate formats. The system integrates a 16-lens array with uniform Köhler illumination, a CMOS-FPGA acquisition module, and a motorized stage that synchronously translates the optical assembly relative to the well plate. During operation, ComplexEye captures one image per well every 8 seconds, producing time-resolved sequences suitable for migration analysis across 96- and 384-well plates. Full details on optical design, electronics, and performance characteristics are available in the original ComplexEye publication^12^.

### Dataset

We evaluated FlowRoI on a dataset consisting of six ComplexEye videos comprising a total of 2628 2D images. These videos depict primary human neutrophils isolated from peripheral blood, and the dataset is publicly available online^17^. Additional information regarding sample preparation and ethical approvals is provided in the ComplexEye paper^12^, which includes the corresponding ethics declarations and informed consent. All images were annotated using a semi-automatic, expert-in-the-loop procedure. Initial instance-level segmentation masks were generated with Cellpose-SAM and subsequently reviewed and corrected by a human expert, resulting in unique instance labels for each individual cell in each frame.

This study used publicly available^17^, de-identified data and did not involve new human participant recruitment. Therefore, ethical approval and informed consent were not required. The original data collection was conducted in accordance with relevant ethical guidelines by the data providers^12^.

### Overall framework

**Fig. 10:**
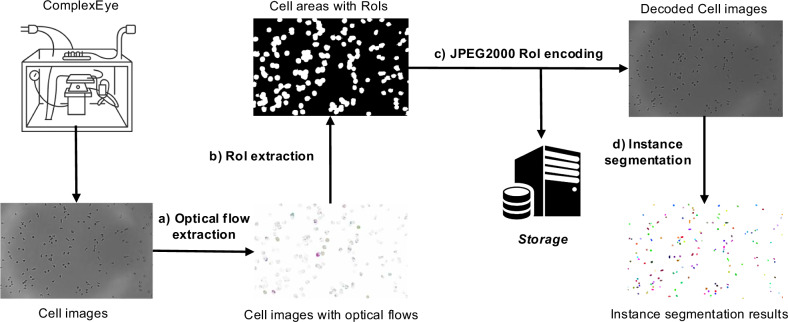
Framework of FlowRoI for high-throughput image compression in immune cell migration analysis. **a** Optical flow extraction from cell images. **b** RoI extraction based on motion estimation. **c** JPEG2000 RoI encoding for storage and transmission. **d** Instance segmentation applied to decoded images to recover cell-level information.

For downstream migration analysis, precise localization of cells within each frame is essential. However, most pixels in an immune cell image correspond to background regions that do not contribute meaningful biological information. The core idea of FlowRoI is to identify and isolate these task-relevant cellular regions and allocate higher encoding priority to them during compression. The overall framework of FlowRoI is illustrated in Fig. 10 and consists of four main stages. In the first stage, optical flow is computed from the ComplexEye image sequence using pairs of adjacent frames. This captures the local motion patterns associated with migrating cells. In the second stage, regions of interest (RoIs) are derived from the resulting optical-flow field. Here, RoIs correspond to cell regions, which are critical for downstream tasks such as semantic segmentation, instance segmentation, cell tracking, and motion quantification. In the third stage, JPEG2000 RoI encoding is applied. RoI pixels are encoded with higher priority—meaning lower compression—while background pixels receive lower priority. This differential treatment allows the compressed bitstream to retain high fidelity in biologically meaningful regions while substantially reducing overall data size. The compressed output can be stored or transmitted efficiently. In the final stage, the encoded images are decoded and subsequently used for downstream analysis. To assess the effectiveness of FlowRoI, we employ cell instance segmentation as a representative downstream task. Accurate instance segmentation is a prerequisite for many migration-analysis pipelines, including cell tracking, trajectory estimation, motility analysis, and morphology quantification.

### Optical flow extraction

Optical-flow extraction consists of three sequential refinement stages: denoising, stabilization, and flow estimation. Image noise is reduced using a two-step denoising strategy that combines median filtering and bilateral filtering to suppress high-frequency noise while preserving cellular boundaries. To correct global translational drift introduced during image acquisition, each frame is then registered to its predecessor via phase-correlation-based alignment. After denoising and alignment, pixel-wise motion is estimated using the dense Farnebäck optical-flow algorithm^21^, which produces displacement vectors describing local cellular and subcellular motion. This pipeline ensures that optical flow is computed from noise-reduced and temporally aligned image pairs, yielding stable and accurate flow fields for subsequent RoI extraction.

### Region of interest extraction

RoI extraction from the flow field follows a saliency-based segmentation procedure. A motion-saliency map is first constructed by combining flow magnitude and spatial gradients with a weighting factor of 0.7. This value was empirically selected in preliminary experiments to balance motion sensitivity and spatial stability under the evaluated setting. This enhances regions exhibiting coherent local motion while suppressing isolated noise responses. The saliency map is then normalized to highlight dynamically active regions across the sequence. Pixels exceeding a quantile-based threshold (the RoI threshold) are selected to generate an initial binary mask. Morphological opening and closing are applied to remove small spurious components and to fill small gaps. Further refinement is performed by removing residual small connected components based on area criteria to ensure that only sufficiently large, biologically meaningful regions are retained. When motion between adjacent frames is weak, FlowRoI optionally incorporates RoI masks from neighboring frames, producing a more stable and complete mask. The overall process yields a clean and compact binary RoI mask that delineates motion-sensitive regions corresponding to the majority of cells.

### JPEG2000 RoI encoding and Instance segmentation

Following optical-flow estimation and RoI extraction, the original image and corresponding RoI mask are encoded using JPEG2000^22^. JPEG2000 RoI compression allows different compression strengths to be applied to different image regions, preserving higher fidelity in important areas while retaining strong overall compression. Among the available RoI tools in the JPEG2000 standard, FlowRoI employs the scaling-based RoI method, in which a user-controlled scaling factor specifies the relative importance of RoI pixels. For downstream evaluation, decoded images are processed using a modern instance segmentation network.

### Hyperparameters

FlowRoI includes five hyperparameters, summarized in Table 2. Denoising is optional and may be enabled or disabled depending on the imaging setup. Although denoising may remove a small number of very low-contrast cells, our experiments indicate that these cells occur infrequently and are typically too challenging even for state-of-the-art deep networks to detect reliably. Users may adjust this parameter based on their specific data characteristics. The RoI threshold controls the proportion of high-saliency pixels retained as RoI. For example, a threshold of 0.2 selects the top 20% of saliency values. The adjacent factor determines whether RoI masks from neighboring frames are incorporated to stabilize the current mask. The remaining hyperparameters—the scaling factor and compression rate—correspond to JPEG2000 RoI settings, where a larger scaling factor increases RoI priority during encoding and a larger compression rate results in stronger compression and smaller file size. Overall, the evaluated hyperparameters exhibit relatively stable performance trends within moderate parameter ranges. Although interactions among parameters (e.g., RoI threshold and scaling factor) may further influence compression behavior, a comprehensive multidimensional hyperparameter optimization study is beyond the scope of the present work.

**Table 2:** Hyperparameters in FlowRoI and their illustrations

Parameter name	Illustration	Values
Denoise	Whether denoise is performed in optical flow extraction	On or off
RoI threshold	The proportion of optical-flow pixels retained for RoI extraction	(0, 1)
Adjacent factor	The number of adjacent masks considered for the ensemble used in RoI extraction	0,1,2 or more
Scaling factor	The relative importance of the RoI over the background in JPEG2000 RoI encoding	[1, 10]
Compression rate	The target compression rate used in JPEG2000 RoI encoding	numbers larger than 1

### Implementation details

FlowRoI was implemented and tested on a laptop equipped with an Intel i7-1255U CPU. For instance, segmentation, we adopted Cellpose-segment anything model (Cellpose-SAM)^18^ and fine-tuned it using 240 annotated images. These training images were selected as a subset of the fully annotated dataset by taking the first 40 frames from each of the six videos, yielding 6 × 40 = 240 images in total. The backbone segment anything model (SAM) model was selected, with a batch size of 2, learning rate of 0.02, and 400 training epochs. Training was performed using the PyTorch framework^23^ on a single NVIDIA RTX A100 GPU. During inference, all configurations used identical model parameters. All algorithmic steps and hyperparameters are explicitly described to facilitate independent reimplementation of the proposed framework.

In this study, we use frame-wise JPEG2000^24^ as the primary compression baseline, following common practice in biomedical imaging, where still-image compression is widely used for storage and archival purposes. JPEG2000 was selected because it provides transparent rate-control behavior and native support for RoI coding, which allows us to isolate and evaluate the contribution of the proposed motion-driven RoI extraction strategy under a controlled compression setting. Our goal in this work is not to establish a comprehensive comparison among compression codecs, but rather to evaluate whether motion-derived RoI maps can improve task-relevant information preservation under a controlled JPEG2000 RoI-coding setting. Accordingly, we do not claim that modern video codecs are ineffective for microscopy compression. Instead, FlowRoI should be interpreted as a lightweight, training-free, and codec-agnostic RoI extraction mechanism that could potentially be integrated with other compression frameworks in future work.

Note that JPEG2000 RoI coding was implemented using the standard Maxshift mechanism with a scaling factor, which applies only when RoIs are enabled.

For instance-segmentation evaluation, we report precision, recall, and F1-score at an IoU threshold of 0.5. We use precision(IoU = 0.5) rather than average precision, as our evaluation is performed at a single IoU threshold without sweeping across confidence levels. For each frame, true positives (TP), false positives (FP), and false negatives (FN) are computed at IoU = 0.5^18^, from which precision, recall, and F1-score are derived. These metrics are then averaged across all frames in the test set to obtain the final reported values.

For image quality evaluation, peak signal-to-noise ratio (PSNR) is used. PSNR is computed on a per-image basis and then averaged over all test frames. Unless otherwise specified, all reported PSNR values in cell regions are computed exclusively over pixels belonging to expert-annotated cell masks, independent of the RoI masks generated by FlowRoI. In particular, cell regions are defined by expert-annotated instance-segmentation masks, in which each individual cell is manually delineated and assigned a unique instance label. This definition ensures that the evaluation reflects reconstruction fidelity strictly within ground-truth cellular regions, rather than being influenced by the presence, absence, or spatial extent of algorithmically generated RoIs. The test set consists of all frames excluding the first 40 frames from each of the six videos used for training (i.e., 2628–240 = 2388 frames). All test frames are included in the evaluation, and no subset selection or filtering is applied. We note that PSNR in this study is primarily evaluated within expert-annotated cell regions, which reflects reconstruction fidelity in biologically relevant areas. This choice is motivated by the task-driven nature of our work, where downstream analysis focuses on cellular regions rather than background. However, we acknowledge that region-restricted evaluation may favor RoI-based methods such as FlowRoI, as these approaches explicitly prioritize selected regions during compression. Global image quality metrics (e.g., full-image PSNR or SSIM) may provide a more holistic assessment of compression performance across the entire image. In this work, our goal is to evaluate compression performance with respect to task-relevant information preservation. A comprehensive comparison including global image quality metrics represents an important direction for future work.

### Statistical analysis

For all quantitative evaluations, metrics were first computed independently for each video and then aggregated across the six videos. Results are reported as mean ± standard deviation unless otherwise specified. To assess differences between FlowRoI and JPEG2000 under matched settings, paired Wilcoxon signed-rank tests were performed across videos. A two-sided *p* < 0.05 was considered statistically significant. Because frames within the same video sequence are temporally correlated, statistical comparisons were performed at the video level rather than the individual-frame level.

## Supplementary information

Supplementary Information

## Data Availability

The dataset used in this study is available at the following URL: https://www.ebi.ac.uk/biostudies/bioimages/studies/S-BIAD2196.

## Code availability

The code used in this study is available at https://github.com/MMV-Lab/FlowROI.
