# Supplementary material for: FlowRoI: fast optical-flow-based Roi extraction for high-throughput immune cell image compression
Source: Npj Imaging. 2026 Jul 11;4:47. doi: 10.1038/s44303-026-00178-3 (PMC13389428; doi:10.1038/s44303-026-00178-3)
Supplement: Supplementary file 1 — Supplementary Information [file 44303_2026_178_MOESM1_ESM.pdf]

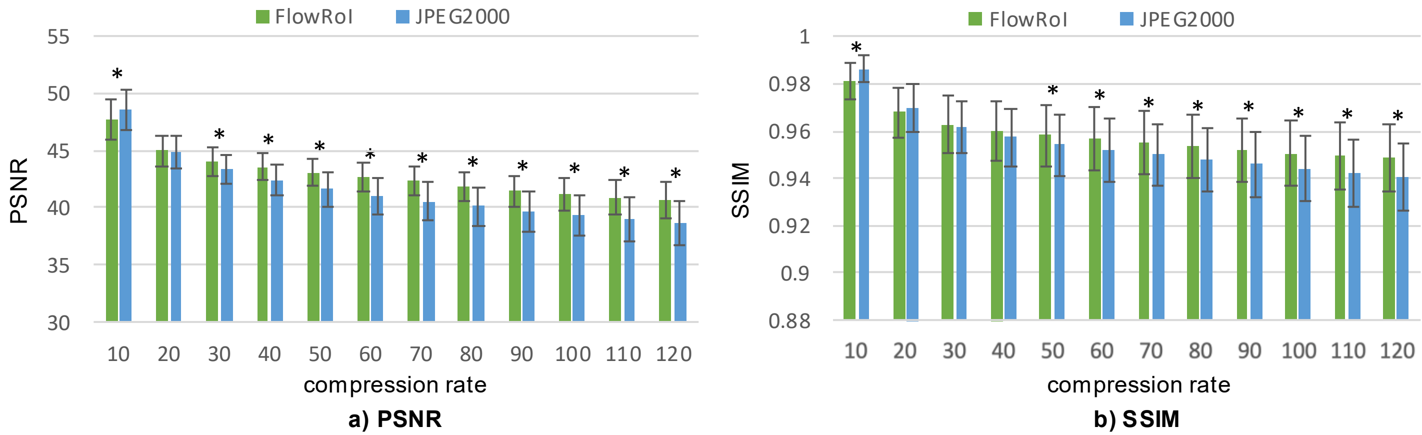

Figure S1. Comparison of FlowRoI and JPEG2000 in terms of full-image reconstruction quality across different compression rates. a Full-image PSNR comparison. b Full-image SSIM comparison. At low compression settings, JPEG2000 may achieve slightly higher global reconstruction quality because sufficient bitrate is available for both foreground and background regions without strong RoI prioritization. Bars indicate mean performance across the six evaluated videos, and error bars represent standard deviation. Statistical significance between FlowRoI and JPEG2000 was assessed using paired Wilcoxon signed-rank tests across videos (*$p < 0.05$).
